# The Role of Epigenetics in Evolution: The Extended Synthesis

**DOI:** 10.1155/2012/286164

**Published:** 2012-03-05

**Authors:** Aaron W. Schrey, Christina L. Richards, Victoria Meller, Vincent Sollars, Douglas M. Ruden

**Affiliations:** ^1^Department of Integrative Biology, University of South Florida, Tampa, FL 33620, USA; ^2^Department of Biochemistry and Molecular Biology, Wayne State University, Detroit, MI 48201, USA; ^3^Department of Biochemistry and Microbiology, Marshall University, Huntington, WV 25755, USA; ^4^Institute of Environmental Health Sciences, C. S. Mott Center for Human Health & Development, Wayne State University, Detroit, MI 48201, USA

Evolutionary biology is currently experiencing an emergence of several research topics that transcend the boundaries of the Modern Synthesis, which was the last major conceptual integration in evolutionary biology [1]. The Modern Synthesis used the concepts of population genetics to integrate Mendelian genetics with evolution by natural selection [2]. Pigliucci [3, and citations within] identified several major areas of innovation that transcend the Modern Synthesis: epigenetics, evolvability, phenotypic plasticity, evolution on adaptive landscapes, evolutionary developmental biology, and systems biology. Integrating these new ideas with the Modern Synthesis will form a new conceptual framework of evolution, which they termed the Extended Synthesis, as it will extend, rather than refute, the Modern Synthesis [3]. This subject has been the focus of much recent work, and an excellent description is provided in the book *Evolution—The Extended Synthesis* [2].

Epigenetics, one of the emerging areas in the Extended Synthesis, is the focus of this special issue. The importance of epigenetics has long been appreciated at the molecular level (e.g., its role in cell determination and self-recognition). However, the role of epigenetics in evolution and ecology is a more recent focus. Epigenetics has expanded to the study of heritable changes in gene expression and function without alterations in the DNA sequence [4], or the study of stably heritable phenotypes that occur without alterations in DNA sequence [5]. Epigenetic mechanisms interact with genetic, physiological, and morphological systems and may be an important component of organism-environment interactions [6, 7]. Some epigenetic characters can be stably transmitted across generations [8–11]. Thus, epigenetics has a mechanism of heredity that was not considered in the framework of the Modern Synthesis [2]. Epigenetic mechanisms may play critical roles in phenotypic plasticity [12, 13], soft inheritance [4, 14], an individual's response to environmental stressors [6, 8], invasive species biology [15], and conservation biology [16]. Understanding epigenetics will likely provide insights into individual and population processes at both ecological and evolutionary time scales [6, 7, 17–19].

DNA methylation, the most studied molecular epigenetic mechanism [20], is active in DNA imprinting [21], X-inactivation [22], restructuring the genome in response to polyploidy caused by hybridization [23], silencing transposable elements [21], and in response to environmental stressors [8]. DNA methylation is a source of interindividual phenotypic variation [10] and has been shown to cause phenotypic variation in flower shape and fruit pigmentation [24, 25], mouse tail shape, adult body size and coat color [26, 27], and numerous traits differentiating queen and worker honeybees [28].

Epigenetic variation in DNA methylation can provide an evolutionarily and ecologically important source of phenotypic variation among individuals. The violet (*Viola cazorlensis*) has a high level of interindividual DNA methylation variation that differentiated populations from southeastern Spain [29], and variation among individuals was related to the amount of damage caused by herbivory [30]. The invasive Japanese knotweed (*Fallopia japonica* and *F. x. bohemica*) has significant differences in DNA methylation among populations from the northeastern United States [31, 32], and a portion of the variation could be attributed to different habitats. Allopolyploid orchids (*Dactylorhiza majalis* s.str, *D. traunsteineri* s.l., and *D. ebudensis*) have variation in DNA methylation that was significantly related to environmental variables [33]. Genetically identical dandelion (*Taraxacum officinale*) plants develop variation in DNA methylation in response to stressors, and many of these changes are stably inherited in the next generation [8]. Also, house sparrows (*Passer domesticus*) from North America and Africa introduced into Europe have a higher level of variation in DNA methylation compared with these birds in their native environments, which suggests that DNA methylation may compensate for the decreased genetic variation caused by introduction into a new environment [34].

In this issue, Castonguay and Angers discuss how epigenetic mechanisms are particularly important in asexual organisms, specifically the asexual hybrid fish *Chrosomus eos-neogaeus*, since epigenetic variation allows for phenotypic variation in otherwise genetically identical individuals. Similarly, Flatscher et al. discuss approaches to disentangle the role of DNA-sequence-based and epigenetic polymorphisms in the process of speciation in the *Heliosperma pusillum* and allied taxa (Caryophyllaceae).

Although DNA methylation is the most well-studied mechanism in the context of ecology and evolution, several studies have investigated other epigenetic mechanisms. Histone modifications, small and long noncoding RNAs, and genome structure can regulate gene expression and contribute to phenotypic variation in diverse taxa [35]. In this issue, Bozzetti et al. discuss the role of the *crystal-Stellate *modifiers, which indicate the importance of piRNA pathways in defense of genome integrity against transposons and other repetitive elements in the gonads and are relevant to evolutionary canalization mechanisms. Wells et al. review different mechanism in which modification of the histone H4 tail modulates gene expression for dosage compensation between sex chromosomes and autosomes and between sexes.

Areas of epigenetics outside of DNA methylation and histone modifications are also discussed in this issue. Apte and Meller review the role of homologue pairing in the transmission of information in flies and mammals and show how communication between homologues affects genome regulation in both taxa. Also, Ferree and Prasad discuss the impact highly repetitive, noncoding satellites have on chromosome segregation at different developmental stages and through distinct cellular mechanisms and note their effect on postzygotic reproductive isolation.

While a great deal of work remains, epigenetics has already proven to be very promising in evolutionary biology. Empirical studies that demonstrate the role epigenetic variation has in ecology and evolution will help answer some of the major questions in evolutionary epigenetics, and these empirical studies will allow a development and refinement of a foundational theory of evolutionary epigenetics. In this issue, Maggert cautions about the potential to dilute epigenetics by confounding true cases of heritable nonsequence information with possibly trivial modes of gene regulation, while Bateson argues how the experience of an individual affects the evolutionary potential of its offspring through epigenetic effects. These two papers in particular indicate that it is important to consider if stable inheritance of the epigenetically derived character is a requirement for evolutionary epigenetics. Alternatively, could the Extended Synthesis integrate epigenetic mechanisms that generate variation and respond to the environment, even if the specific changes are not inherited? In certain cases, the presence of an additional source of variation may be most important. In others, the stable transmission of a particular epigenetic state may be important. Ultimately, the increased phenotypic potential of a genotype via epigenetic mechanisms, which in some cases may be inherited, must be incorporated into the evolutionary theory.

Before presenting the papers of this special issue, we would like to alert the reader to a second special issue planned for Genetics Research International: *The Epigenetics of Emerging and Nonmodel Organisms* (edited by Vett Loyd et al.). Together, these two special issues introduce the reader to the importance of epigenetics in evolution and developmental biology.

*Aaron W. Schrey* *Aaron W. Schrey* *Christina L. Richards* *Christina L. Richards* *Victoria Meller* *Victoria Meller* *Vincent Sollars* *Vincent Sollars* *Douglas M. Ruden* *Douglas M. Ruden*
